# Dichloroacetate, the Pyruvate Dehydrogenase Complex and the Modulation of mESC Pluripotency

**DOI:** 10.1371/journal.pone.0131663

**Published:** 2015-07-06

**Authors:** Ana Sofia Rodrigues, Marcelo Correia, Andreia Gomes, Sandro L. Pereira, Tânia Perestrelo, Maria Inês Sousa, João Ramalho-Santos

**Affiliations:** 1 PhD Programme in Experimental Biology and Biomedicine, CNC—Center for Neuroscience and Cell Biology, University of Coimbra, Coimbra, Portugal; 2 CNC—Center for Neuroscience and Cell Biology, University of Coimbra, Coimbra, Portugal; 3 Institute for Interdisciplinary Research (IIIUC), University of Coimbra, Coimbra, Portugal; 4 Biocant—Center of Innovation in Biotechnology, Cantanhede, Portugal; 5 Department of Life Sciences, University of Coimbra, Coimbra, Portugal; University of Nebraska Medical Center, UNITED STATES

## Abstract

**Introduction:**

The pyruvate dehydrogenase (PDH) complex is localized in the mitochondrial matrix catalyzing the irreversible decarboxylation of pyruvate to acetyl-CoA and NADH. For proper complex regulation the E1-α subunit functions as an on/off switch regulated by phosphorylation/dephosphorylation. In different cell types one of the four-pyruvate dehydrogenase kinase isoforms (PDHK1-4) can phosphorylate this subunit leading to PDH inactivation. Our previous results with human Embryonic Stem Cells (hESC), suggested that PDHK could be a key regulator in the metabolic profile of pluripotent cells, as it is upregulated in pluripotent stem cells. Therefore, we wondered if metabolic modulation, via inexpensive pharmacological inhibition of PDHK, could impact metabolism and pluripotency.

**Methods/Results:**

In order to assess the importance of the PDH cycle in mouse Embryonic Stem Cells (mESC), we incubated cells with the PDHK inhibitor dichloroacetate (DCA) and observed that in its presence ESC started to differentiate. Changes in mitochondrial function and proliferation potential were also found and protein levels for PDH (both phosphorylated and non-phosphorylated) and PDHK1 were monitored. Interestingly, we were also able to describe a possible pathway that involves Hif-1α and p53 during DCA-induced loss of pluripotency. Results with ESCs treated with DCA were comparable to those obtained for cells grown without Leukemia Inhibitor Factor (LIF), used in this case as a positive control for differentiation.

**Conclusions:**

DCA negatively affects ESC pluripotency by changing cell metabolism and elements related to the PDH cycle, suggesting that PDHK could function as a possible metabolic gatekeeper in ESC, and may be a good target to modulate metabolism and differentiation. Although further molecular biology-based experiments are required, our data suggests that inactive PDH favors pluripotency and that ESC have similar strategies as cancer cells to maintain a glycolytic profile, by using some of the signaling pathways found in the latter cells.

## Introduction

Rapidly proliferating cells such as cancer or embryonic stem cells (ESCs) rely on a characteristic intermediary metabolism to, not only fulfill all their bioenergetic demands, but also provide the necessary building blocks for biosynthesis, in order to support proliferation [1]. It has been shown that hypoxia and mitochondrial inhibition are beneficial for ESC pluripotency maintenance [2–5] and that somatic cell reprogramming requires a metabolic shift to glycolysis before activation of the endogenous pluripotency genes can take place [1,6,7]. Under normoxic conditions glycolysis is defined as the conversion of glucose to pyruvate that can be further metabolized in the mitochondria via the activity of pyruvate dehydrogenase (PDH), which converts pyruvate to acetyl-CoA [1]. The PDH complex is localized in the mitochondrial matrix, and catalyzes the irreversible decarboxylation of pyruvate to acetyl-CoA and NADH, with an E1-α subunit that functions as an on/off switch, regulated by phosphorylation/dephosphorylation events. One of the existing four-pyruvate dehydrogenase kinase isoforms (PDHK1-4) can phosphorylate this subunit, thus causing inactivation of PDH. Interestingly, in pluripotent stem cells, PDHK is upregulated, phosphorylating PDH and consequently inactivating it [7,8]. As a logical outcome pyruvate obtained from glycolysis cannot be transformed into acetyl-CoA, and instead is converted to lactate, maintaining the glycolytic profile of proliferating cells. Modulation of PDHK activity can be accomplished by adding pyruvate to the culture medium or the chemical compound dichloroacetic acid (DCA), which inhibits the enzyme [9–12]. The emergent role of PDHK in regulating PDH status in cancer, in parallel with our previous results, raises the possibility that modulating the PDH cycle could have an impact on metabolism and pluripotency, and possibly be used to modulate ESC differentiation. Intriguingly, PDHK has already been suggested as a specific target in cancer cells and some of its inhibitors, such as DCA, have being considered for possible therapeutic purposes [13,14]. Indeed, DCA is known for inhibiting all PDHK isoforms and it as been used in clinical trials for several types of tumors (lung, endometrial and breast cancer[12]) and other clinical conditions such as type II diabetes [15], congestive heart failure and congenital mitochondrial diseases [12] due to side effect of lowering lactate levels by activating the PDH complex. DCA is a small molecule of 150 Da that penetrates easily into the cell and activates PDH in a dose dependent manner. It has been described that DCA leads to an increase in ROS production due to a shift in metabolism [13,14].

Therefore, we aimed to us a simple pharmacological approach to test if PDH could indeed be crucial for pluripotency, and if some of the metabolic regulatory pathways, found in cancer cells are also present in ESCs, which could constitute a link between cancer proliferation and stem cell pluripotency. Metabolic regulators such as HIF-1α, and PDHKII have been implicated not only in cancer [16,17] but also in induced pluripotency [7], so we wondered if this type of regulatory network is also present in ESCs. Given that in cancer other known players, such as p53 and HIF-1α, regulate metabolism, we decided to clarify if they could also play a role in ESC metabolism/pluripotency status, once more highlighting the possible similarities between cancer cells and ESCs.

In order to address this question we treated mESCs with DCA and grew cells without the essential pluripotency mediator Leukemia inhibitor factor (LIF) as a positive control for differentiation. Overall we present a putative target for metabolic modulation with consequences for pluripotency and shed some light into some possible metabolic regulators in mESCs. We also address the possibility that a simple and inexpensive pharmacologically based metabolic switch may be useful in order to control pluripotent stem cell fate.

## Material and Methods

### Cell culture conditions and Experimental design for Dichloroacetic acid (DCA)

The mouse embryonic stem cell line E14Tg2a was kindly provided by Miguel Ramalho-Santos (University of California, San Francisco, USA) and characterized elsewhere [18,19]. Cells were maintained in feeder free conditions using Knockout-DMEM media (Gibco LIFe Technologies) supplemented with 15% of KSR (Knockout serum replacement—Gibco LIFe Technologies), 1% of MEM Non-Essential Amino acids (Sigma-Aldrich), 1% Penicillin/Streptomycin, 1% L-glutamine (2mM) (both from Gibco LIFe Technologies) and β-Mercaptoethanol (Sigma-Aldrich). In order to maintain pluripotency Leukemia inhibiting factor (LIF) (ESGRO Millipore) was added at a final concentration 10U/L. Media was changed every 24hours and cells were maintained at 37°C, 20%O2 and 5% CO2. E14Tg2a mESC were passaged when the right confluence was achieved, usually two or three days after platting. Briefly, 0.1% gelatin (Sigma-Aldrich) was added to plates and allowed to coat for ten minutes at 37°C. Afterwards, the excess was removed and supplemented Knockout-DMEM media was added. Cells were plated at a final density of 5000cells/cm for all experimental conditions: cells in control conditions, in the absence of LIF and in the presence of LIF plus two different DCA (Sigma-Aldrich) concentrations (3 and 5 mM). DCA was freshly prepared and added every 24h and experiments were conducted after 84h of incubation.

### Viability

In order to monitor cell viability, the LIVE/DEAD Kit (Invitrogen) was used according to manufacturer’s instructions. The Kit consists of two DNA binding fluorescent dyes: SYBR 14 which is membrane permeable staining the nucleus green for all cells, and PI (propidium iodide). PI will only enter cells with compromised membrane integrity, thus staining the nucleus of dead cells red. Briefly, cells were collected at the 60 h time point by enzymatic dissociation with accutase (Gibco LIFe Technologies), and centrifuged for 5 min at 1200rpm. The pellet was ressuspended in D-PBS and 6μM of SYBR 14 and 0.48mM of PI were added to the cell suspension that was then incubated for 20 min at 37°C, 20%O2 and 5% CO2. Viability was assessed with a fluorescent microscope, by counting 100 cells per condition; green cells without red fluorescence were counted as live cells and a cell with both stains was considered dead.

### High Resolution enzyme-linked immunosorbent assay (ELISA)

To assess the pluripotency status of our experimental conditions, mESCs were plated in a 24 well plate,rinsed with D-PBS at the collection time point and then fixed for 30 min with 4% paraformaldehyde (PFA). A permeabilization step was performed with 1% Triton X-100 and cells incubated with a 3% H2O2 solution for 5 min to stop endogenous peroxidase activity. Cells were blocked for 1 h at room temperature with 3% BSA, 0.25 Triton X-100 in PBS. Incubation with primary antibodies against Oct4, Nanog and Gapdh (1:500 was the dilution for all antibodies) occurred overnight at 4°C. Prior to incubation with the proper HRP-conjugated secondary antibodies (1:1000) cells were washed 3 times for 5 min with D-PBS. To detect HRP activity, cells were incubated for 10 min with the chromogenic substrate tetramethylbenzidine (TMB-Sigma-Aldrich). The reaction was stopped with the addition of 1M H2SO4 and color quantification was done at 450 nm in a microplate reader [20]. In order to normalize the results total mass quantification was performed using sulforhodamineB (SRB, Sigma-Aldrich) [21].

### Thiazolyl Blue Tetrazolium Bromide (MTT) assay

MTT was reconstituted according to the manufacturers’ instructions (Sigma-Aldrich) and was used at a final concentration of 0.5mg/ml. This assay is routinely used as an assay for cell proliferation/metabolic activity. This is due to the fact that MTT is reduced by cellular dehydrogenases (using both NADPH and NADH) present in the cells, and this will produce violet formazan crystals that are soluble in acidified isopropanol [22]. Cells were plated and incubated for 60 h and media was changed so that the assay did not take place in the presence of DCA. Formazan crystals (violet) formed after a 5 h incubation at 37°C, 20%O2 and 5% CO2 and were solubilized with 300 μl of isopropanol with HCl 0.04M. Violet color intensity was measured colorimetrically at 570 nm. Raw data was normalized to total cell number for each condition and then metabolic activity was normalized to the control.

### Alkaline phosphatase assay

It is well accepted that stem cells that are self-renewing and pluripotent present high levels of alkaline phosphatase (AP) [23]. As a first approach to monitor pluripotency status the AP assay (using the Alkaline Phosphatase Detection Kit from Millipore) was performed for each experimental condition following the protocol provided by Millipore. Briefly, cells were cultured in a 24 well plate for 60h, media was removed and cells were fixed with 4% paraformaldehyde for one minute. Cells were washed and the alkaline phosphatase reagent (prepared according to the manufacturers’ instructions) was added. After a 20 min incubation at room temperature (RT) in the dark the reagent was removed and D-PBS was added. Colonies were counted using an optical microscope; Colonies presenting red color, typical of the AP positive staining, were counted as positively marked, while colonies with no red staining were counted as AP negative. All colonies in the 24 wells were counted and results normalized to 100%. Experiments were performed in duplicates for all experimental conditions.

### Flow cytometry

Analysis by flow cytometry (BD FACSCalibur) involved 20000 gated cells acquired/analyzed per condition with the Cell Quest Pro Acquisition software (BD Biosciences).

Mitochondrial membrane potential (MMP) was analyzed using tetramethylrhodamine methyl (TMRM-Invitrogen) [24–26] a lipophilic cationic fluorescent dye that, due to its positive charge, accumulates in the mitochondria according to membrane potential. Cells were incubated with 20μM of TMRM for 20 min at 37°C, 20%O2 and 5% CO2 in the dark in 1ml of D-PBS. Afterwards cells were centrifuged to remove excess TMRM and pellets were ressuspended with 500μl of PBS, kept on ice and analyzed. In order to define the proper gates for an accurate analysis we used cells without TMRM as a blank control and TMRM labeled cells incubated with 250 μM of CCCP, a potent mitochondrial uncoupler as a positive control.

To evaluate the intracellular amounts of the superoxide anion we used MitoSOX Red (Molecular Probes) that fluoresces after selectively reacting with superoxide in mitochondria. MitoSOX Red was prepared according to the manufacturers’ instructions and cells were incubated for 30 min at 37°C in the dark with a final concentration of 3μM of the probe. To properly define the analysis gates we used cells without the probe as a negative control and cells that were incubated with Antimycin A (100μM) as a positive control, given the property of this substance as a potent mitochondrial complex III inhibitor and ROS inducer [27–29].

To monitor effects on cell proliferation we examined the expression of the proliferating cell nuclear antigen (PCNA), highly expressed in rapidly proliferating cells [30]. Cells were fixed with 70% ethanol and stored overnight at -20°C, then subjected to an acidic denaturation step with 2N HCl and washed. Afterwards cells were incubated for 1h with primary antibody against PCNA (1:100), washed with D-PBS, FITC-conjugated secondary antibody was added (1:100) and cells incubated for 1h in the dark [31]. As controls we used cells without antibodies (to access auto-fluorescence), as well as cells incubated only with the primary antibody and cells incubated only with the secondary antibody.

### Adenine nucleotide content analysis by High Performance liquid chromatography (HPLC)

The protocol was as previously described [8]. Samples were stored at -80°C until assayed by separation in a reverse-phase HPLC using a Beckman-System Gold. The detection wavelength was 254 nm, and the column used was a LiChrospher 100 RP-18 (5 μM, Merck). The elution buffer was composed by 100mM phosphate buffer (pH 6.5) and supplemented with 1% methanol. Retention times were determined using standards for ATP, ADP and AMP (Sigma-Aldrich): Adenylate Energy Charge was calculated according to the following formula: ATP+0.5xADP/(ATP+ADP+AMP) [32,33].

### Total RNA isolation, DNA cleanup, cDNA synthesis and RT-PCR

RNA isolation and DNA cleanup was performed as described [4]. Following RNA collection, concentration as well as RNA quality was determined using NanoDrop 2000 (Thermo Scientific) and samples presenting a 260/280 ratio under 1.8 were discarded. Samples of total RNA were stored at -80°C until use [4]. cDNA was obtained using the iScript cDNA Synthesis Kit from Bio Rad according to the protocol established from the manufacturer. Afterwards, samples were placed in the thermal cycler (S1000 Thermal Cycler) programmed with the reaction protocol provided by the manufacturer. RT-PCR was performed to quantify gene expression for Oct4; Nanog; Gapdh; Hexokinase II and Hexokinase I with beta-Actin used as housekeeping gene for data normalization. Primers were obtained from a primer bank database (http://pga.mgh.harvard.edu/primerbank/) and ordered from Integrated DNA Technologies (IDT). SsoFast EvaGreen Supermix (Bio-Rad) was used to perform qRT-PCR analysis according to instructions provided by Bio-Rad. The Glucose array from SABiosciences (Frederick, MD) was used according to the manufacturers’ instructions. Samples were run in CFX96 Touch Real-Time PCR Detection System and mRNA fold change was calculated using the -∆∆Ct method. Clustering was performed using the CFX manager software by Bio-Rad.

### Total Protein Extracts and protein quantification

In order to obtain protein extracts for Western blot analysis mESC were lysed with 100μl of RIPA buffer (Sigma-Aldrich) supplemented with 2mM of phenylmethylsulphonyl fluoride-PMSF (Sigma-Aldrich) and 2x Halt phosphatase inhibitor cocktail (Pierce, Rockford, IL) as described elsewhere [8]. Protein quantification was performed using the Pierce BCA (Bicinchoninic Acid) Protein Assay Kit, following the datasheet protocol. Both samples and calibration curve were determined with duplicates.

### Western Blot protocol

Protein samples for Western blot were prepared by diluting 30μg of protein in Laemmli sample buffer (Bio-Rad) and water, given that the volume of sample buffer had to contain 30μg of protein (water was used to adjust the volume). A total volume of 30μl per sample was prepared and denatured at 95°C in a dry bath. After this step samples were loaded into 12% Acrilamide Tris-HCl gel and electrophoresis was performed in a Mini protean tetra cell Bio-Rad apparatus. After protein size separation in the gel, proteins were blotted into a PDVF membrane (Bio-Rad) and blocked in a solution of 5% powder milk (Bio-Rad) in Tris-Buffered Saline with Tween (TBST). Afterwards, membranes were incubated overnight at 4°C with the primary antibodies: P53 anti-mouse, PDH anti-rabbit, c-MYC anti-rabbit (Cell Signaling Technology, 1:1000), HIF 1 alpha anti-mouse (Thermo Scientific, 1:1000), GAPDH anti-rabbit (Cell Signaling Technology, 1:2000), Hexokinase I anti-mouse and Hexokinase II anti-mouse (Cell Signaling Technology, 1:500) and anti-mouse beta-Actin (Sigma-Aldrich, 1:5000). Membranes were washed and then incubated with the correct HRP-conjugated secondary antibody. Proteins were detected using the Clarity Western ECL Substrate (Bio-Rad) and membranes were developed using the VersaDoc Imaging system (Bio-Rad). Protein quantification was performed using Quantity One software and results were normalized to beta-Actin levels for each condition.

### Statistical Analysis

SPSS Statistics 21.0 (SPSS Inc.) was used to perform statistical analysis. All the raw data was verified for normality and homoscedasticity and accordingly the appropriate parametric tests for paired samples were applied. Given that all the experimental conditions were collected/analyzed at the same time and under the same conditions repeated measure ANOVA was used. Sphericity was assessed and when violated the Greenhouse-Geisser correction was applied before performing any POST-HOC tests. When data violated the normal distribution assumption a Friedman ANOVA was performed. All data is expressed as mean ± standard error of mean (SEM) reflecting the number of performed experiments. Statistical significance was determined at p ≤ 0.05. The p values for the Glucose array were calculated using SABiosciences online software, and statistical significance was determine at p ≤ 0.05.

## Results

### DCA effects on colony morphology, total cell number, viability and proliferation

**Fig 1:**
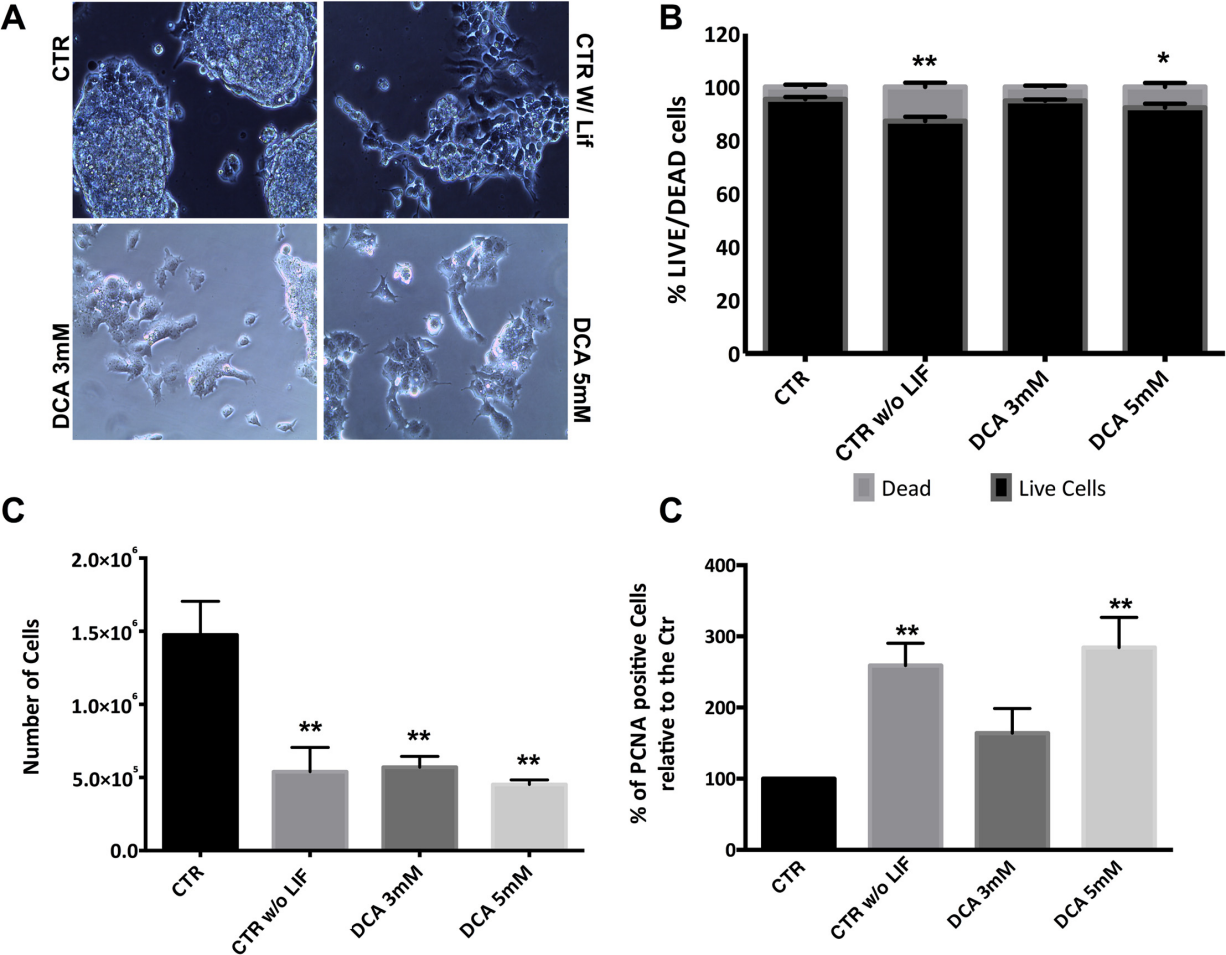
Effect of DCA on morphology, viability, cell number and proliferation. (A) Phase microscopy photographs of ESC colonies in all conditions with a magnification of 200x. Control ESCs (CTR) present tightly packed round colonies with well-defined borders. ESCs cultured in the absence of LIF (CTR w/o LIF) present colonies that are differentiating. ESCs incubated in the presence of LIF plus 3 mM or 5 mM DCA, show high differentiation levels, similar to the differentiation control. (B) Percentage of viable *versus* dead cells. Cells stained green were counted as live, while PI positive cells were counted as dead. 100 cells were counted per condition for a total of n = 8 independent experiments. Results are expressed as means and error bars represent SEM for 30 experiments. (C) Total number of cells counted using a Neubauer chamber prior to collection for ATP and flow cytometry experiments. Results are expressed as means and error bars represent SEM. (D)- Cells in all experimental conditions were stained for PCNA in the cells nucleus and protein levels were assessed by flow cytometry. Results were analyzed in terms of Geometric Mean of Fluorescence for 20000 cells for each condition and are represented as percentage relative to the control. 4 independent experiments were performed. * < 0.05; ** p< 0.01.

Although DCA has been used to show that a glycolytic turnover is necessary to induce pluripotency [6], there are no data regarding its effect on pluripotent stem cells. Fig 1A shows that control (pluripotent) cells are arranged in tightly packed round colonies with well-defined borders. On the other hand, cells growing without LIF (as a differentiation control) present spread out colonies that are not well defined, characteristic of differentiated cells. When exposed to 5mM DCA, and to a lesser extent to 3mM DCA, cells show differentiation levels, similar to the differentiation control, although they were cultured with the pluripotency factor LIF. Next, we assessed cellular viability, and a significant decrease in the percentage of live cells for cells grown without LIF and exposed to the higher concentration of DCA in the presence of LIF was observed (P<0.01 and P<0.05, respectively) (Fig 1B). In parallel with viability results, there is a significant decrease in total cell numbers for all experimental conditions when compared to pluripotent control cells (P<0.01) (Fig 1C). To determine if the differences in cell number could be also due to changes in proliferation, we stained cells for PCNA, which is highly expressed in rapidly proliferating cells [30] (Fig 1D). Results show a significant increase in PCNA levels in cells cultured without LIF (P<0.01) and cells incubated with 5mM DCA plus LIF (P<0.01), suggesting that they are both more proliferative. Overall our experimental conditions are affecting cellular viability and number, but cells maintain proliferation capability.

### DCA affects pluripotency

**Fig 2:**
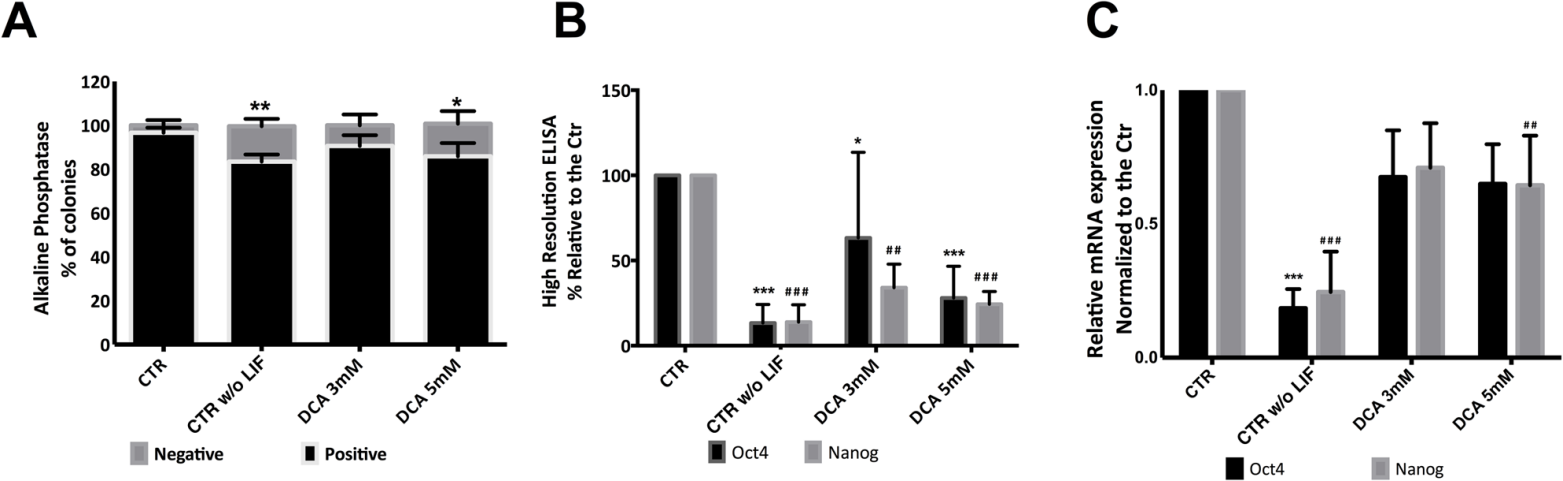
DCA effects on Pluripotency. (A)- Quantification for the alkaline phosphatase assay: colonies stained red were counted as (positive) pluripotent colonies whereas colonies without staining were counted as negative. A total of 10 independent experiments were analyzed for all experimental conditions resulting in a significant negative impact for CTR w/o LIF and DCA 5 mM. (B)- Quantification of pluripotency markers Oct4 and Nanog expression levels using high-resolution ELISA. All experimental conditions present a significant decrease when compared to the control for both pluripotency factors. (C)- qRT-PCR analysis for the Oct4 and Nanog mRNA gene levels results are represented as fold changes normalized to the Control after normalization for endogenous βeta-actin. These results mirror the protein levels regarding CTR w/o LIF and DCA 5 mM. Four independent experiments were performed. All the results are expressed as means and error bars represents SEM. * and # < 0.05; ** and ## p< 0.01; *** and ### p< 0.001 relative to the respective controls.

We next investigated whether DCA affected pluripotency even when ESCs are maintained under pluripotency conditions (with LIF). The alkaline phosphatase (AP) assay (Fig 2A) revealed that cells without LIF have a significant lower number of positive colonies when compared to the control (P<0.01) and the same was true for cells cultured with LIF and 5 mM DCA (P<0.05). When protein levels for the pluripotency markers Oct4 and Nanog were quantified (Fig 2B), a significant decrease was observed for all conditions when compared to the control for both factors. Cells without LIF and those exposed to 5mM DCA had the lowest protein levels (P<0.001) and the same tendency was observed for mRNA levels (Fig 2C). Concomitantly, mRNA levels dropped significantly in absence of LIF (P<0.001) for both genes while in the presence of 5mM DCA plus LIF (Fig 2C) the decrease in Oct4 was more pronounced when compared to Nanog levels, and once more the results for 5 mM DCA paralleled the negative (differentiation) control.

### Mitochondrial Function and metabolic status: Oxidative phosphorylation or glycolysis?

**Fig 3:**
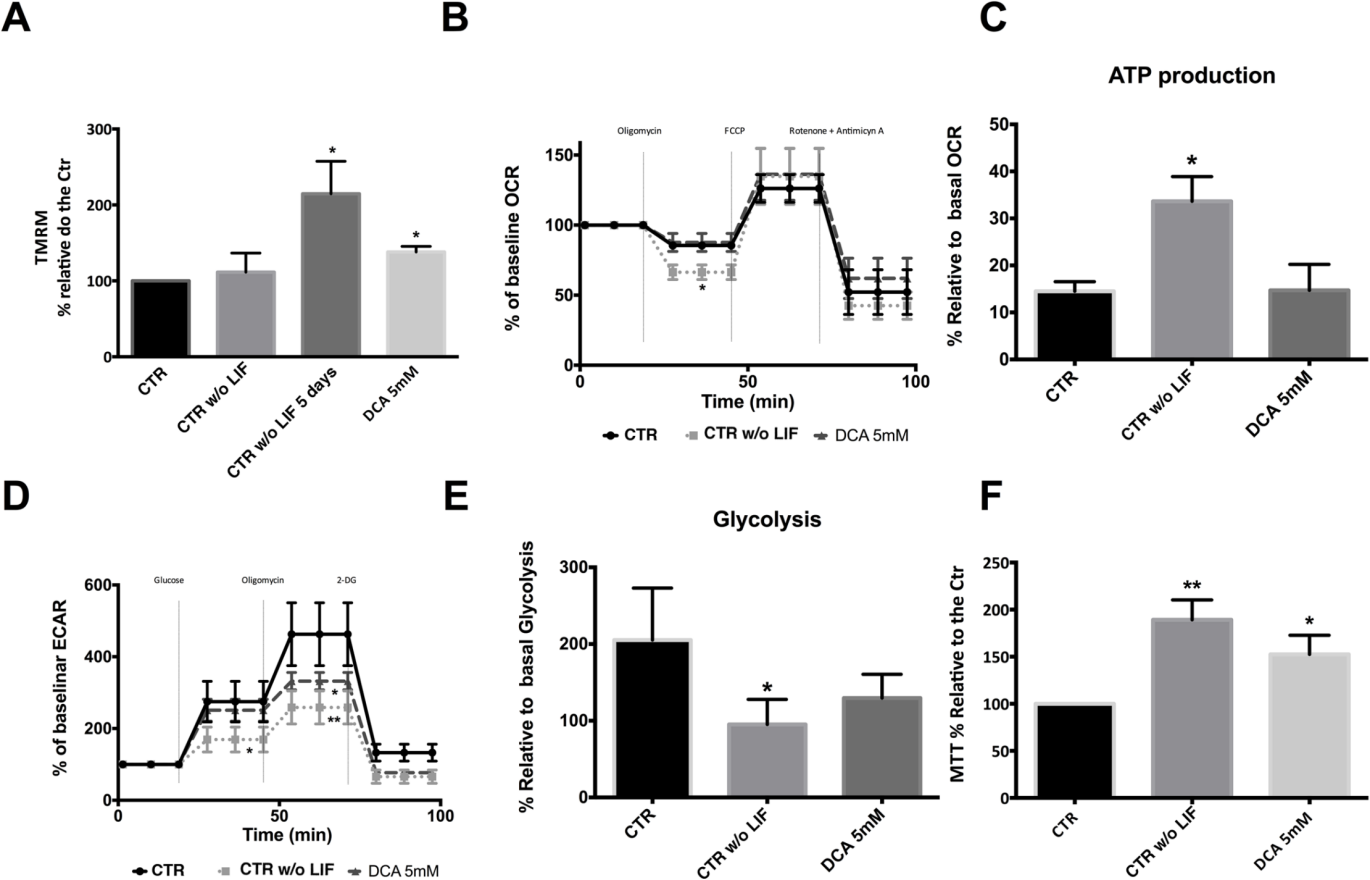
Assessing mitochondrial function. For each flow cytometry experiment results were analyzed in terms of Geometric Mean of Fluorescence for 20000 cells for each condition and are represented as percentage relative to the control. (A)- Quantification of mitochondrial membrane potential (MMP) potential using TMRM by flow cytometry. A significant increase in MMP was observed for CTR w/o LIF as well as for DCA 5 mM. Results are means ± SEM of 4 independent experiments. (B)- Oxygen consumption rate (OCR) was determined using the Seahorse XF24 analyzer. The three mitochondrial inhibitors were sequentially injected (after measurement points, 2, 4, and 6, as indicated) and the final concentrations of each were: oligomycin (1μM); FCCP (300mM); rotenone and antimicyn A (1μM). CTR w/o LIF presented a significant decrease in OCR in the presence of oligomycin. (C)- Determination of the respiration used to drive ATP production under basal conditions following the mathematical calculation: ATP production = Basal–Protocol Leak. This formula determines that CTR w/o LIF cells had higher mitochondrial respiration coupled to ATP production (D)- Measurement of extracellular acidification rate (ECAR) using the Seahorse XF24 analyzer. Cells were incubated in a medium without glucose and ECAR was accessed during consumption of Glucose (added at the third time point at a final concentration of 5mM) and in the presence of oligomycin (1μM added at the sixth point) and 2DG (10mM at the ninth point). At basal conditions, CTR w/o LIF had a lower glycolytic rate when compared to the control. The CTR condition displayed a significant higher glycolytic capacity. (E)- Determination of glucose breakdown to pyruvate using the mathematical calculation: first measurement after glucose injection minus the measurement prior to glucose injection. CTR w/o LIF cells had a significantly lower glycolytic capacity. F)- Results for the MTT assay are presented as percentage of formazan crystals relative to the control. Because the goal was to evaluate cell oxidative status, MTT results were normalized for total cell number. CTR w/o LIF and DCA 5 mM showed a significant increase in the oxidative state. A total of 10 independent experiments were performed. * < 0.05; ** p< 0.01.

Given that DCA has been shown to affect cell metabolism by inhibiting PDHK, with a concomitant cellular shift from glycolysis to oxidative phosphorylation, we next assessed mitochondrial function and metabolic status in ESCs cultured with the drug. Mitochondrial membrane potential (MMP) showed that with increasing concentrations of DCA mitochondria become more hyperpolarized even in the presence of LIF, which leads to a higher MMP for cells cultured in 5 mM DCA (p<0.05), similarly to cells grown without LIF (Fig 3A). To establish if DCA-induced changes could be related to higher mitochondrial activity we determined oxygen (O2) consumption rate (OCR) and glycolysis rate using the Seahorse XF24 extracellular flux analyzer. Taking into account that only exposure to 5mM DCA in the presence of LIF causes significant changes in morphology, proliferation rates, pluripotency status and MMP, we performed the analysis using only this concentration compared to the normal and negative control (cells without LIF). Mitochondrial OCR profiling (Fig 3B) showed that, although we have differences in MMP, these changes are not significant in terms of OCR for cells cultured with 5mM DCA. However, when considering our negative control a significant decrease in OCR in the presence of oligomycin was noted (P<0.05). This inhibitor has the ability to block ATP synthase at the F0 subunit, blocking proton conductance with loss of electron transfer and O2 consumption [8]. A detailed analysis of OCR profile allowed us to determine oxygen consumed for ATP production (Fig 3C), clearly demonstrating that differentiating cells have a higher percentage of OCR coupled to ATP production (P<0.05).

Taking into account that glycolysis and OXPHOS are the two major pathways for ATP production in the cell, and considering that we did not find major changes in the latter, we profiled glycolysis in our experimental conditions. When lactic fermentation linked to glycolysis takes place there is an acidification of the surrounding medium that can be measured directly by the Seahorse XF24 extracellular flux analyzer and reported as extracellular acidification rate (ECAR). Cells were incubated in a medium without glucose so when it is added the glycolytic rate in basal conditions can be calculated. In this case differentiating cells (CTR W/O LIF) have a lower glycolytic rate when compared to the control (P<0.05; Fig 3D). Next, oligomycin was added, forcing cells to rely exclusively on glycolysis for ATP production thus revealing maximum glycolytic capacity. Once more, compared to the control (that displayed higher glycolytic capacity), cells grown without LIF significantly rely less on glycolysis for ATP production (P<0.01). Cells cultured with LIF and 5mM DCA showed an intermediate capacity, and were significantly different from the control (P<0.05). The final inhibitor added was 2-Deoxy-D-Glucose (2DG), which inhibits glycolysis through direct inhibition of Hexokinase (the enzyme that catalyzes the first step in glycolysis) and is used as a control to confirm that the measured ECAR is indeed due to glycolytic activity. Indeed, a severe decrease is observed following 2DG addition for all experimental conditions. ECAR profile analysis revealed that, in agreement with Fig 3D, that cells grown without LIF indeed presents lower glycolysis activity (Fig 3E) when compared to the control mESC (P<0.05).

The data discussed above suggests that control cells are more glycolytic when compared with differentiated and DCA-exposed cells, which are clearly more metabolically active. We confirmed these results with the MTT assay, which, although routinely used as a proliferation test, actually measures the activity of cellular NADPH-dependent oxidoreductase enzymes. Therefore, if we normalize results to the number of cells we cellular oxidative status can be determined. In this case, and in line with previous data, we observed a significant increase for CTR Without LIF (differentiated cells) (P<0.01) and for cells exposed to 5mM DCA in the presence of LIF (P<0.05) (Fig 3F).

### Analysis of possible candidates for metabolic shifts during pluripotency loss

**Fig 4:**
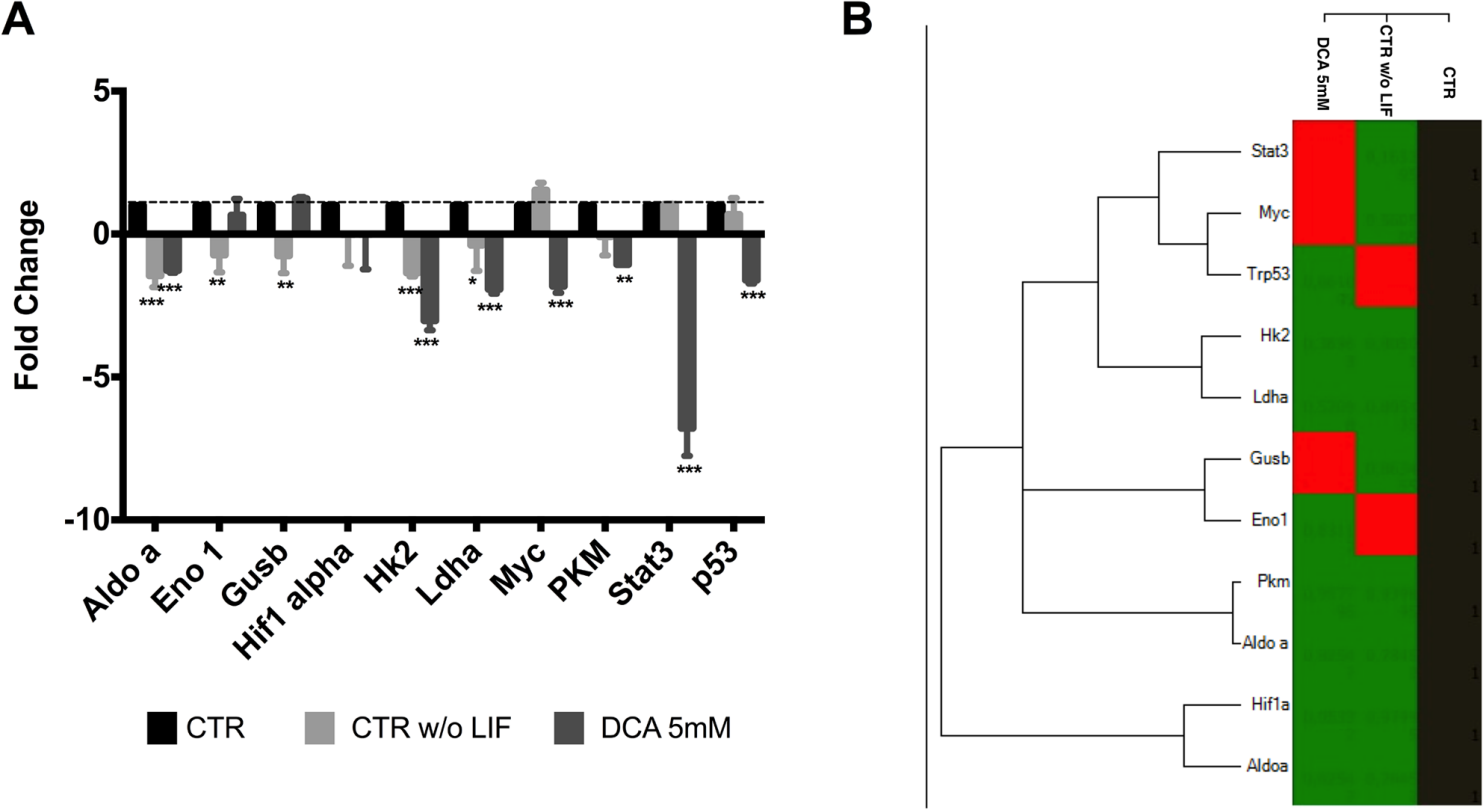
Metabolism-related gene expression in mESC following DCA treatment. (A) Fold changes were calculated for the various genes using the -∆∆Ct method relative to the CTR condition. The values represent means ± SEM of four independent experiments. (B) Heat map of average gene expression represented as log_10_ of Ct values. An increase in gene expression is depicted in red, whereas a decrease in gene expression is represented by the green color. No differences in expression are depicted in black. Clustering was performed using the CFX manager software by Bio-Rad. * < 0.05; ** p< 0.01; ***p< 0.001.

Given that both metabolic changes and a shift towards differentiation were detected when culturing ESCs in the presence of DCA we next tried to identify possible candidate genes that could be involved in this process by designing a custom array. Although focusing on metabolic enzymes, we also decided to include other known players in metabolic shifts such as Hif-1α, c-Myc, p53 and stat3 (Fig 4A). Overall, differentiation decreases gene expression for almost all genes monitored. Interestingly, it is possible to distinguish between differentiation (absence of LIF) and DCA effects in the presence of LIF. DCA seems to try to counteract normal differentiation effects for some genes, notably Enolase 1 (Eno1), Glucuronidase beta (Gusb), c-Myc, Pdhk1, Stat3 and p53. On the other hand, when considering Aldolase a (Aldo a) (P<0.001); Hif-1α; HKII (P<0.001); Ldha (P<0.05 and P<0.001); Pdha1 (P<0.01 and P<0.001) and PKM (P<0.05) DCA seems to have a similar negative impact on these genes when compared to naturally differentiating cells cultured without LIF. Fig 4B supports the idea that DCA is causing a shift towards differentiation given that the clustergram analysis cluster cells exposed to DCA closer to ESCs cultured without LIF.

### Analysis of key elements in metabolic shifts

**Fig 5:**
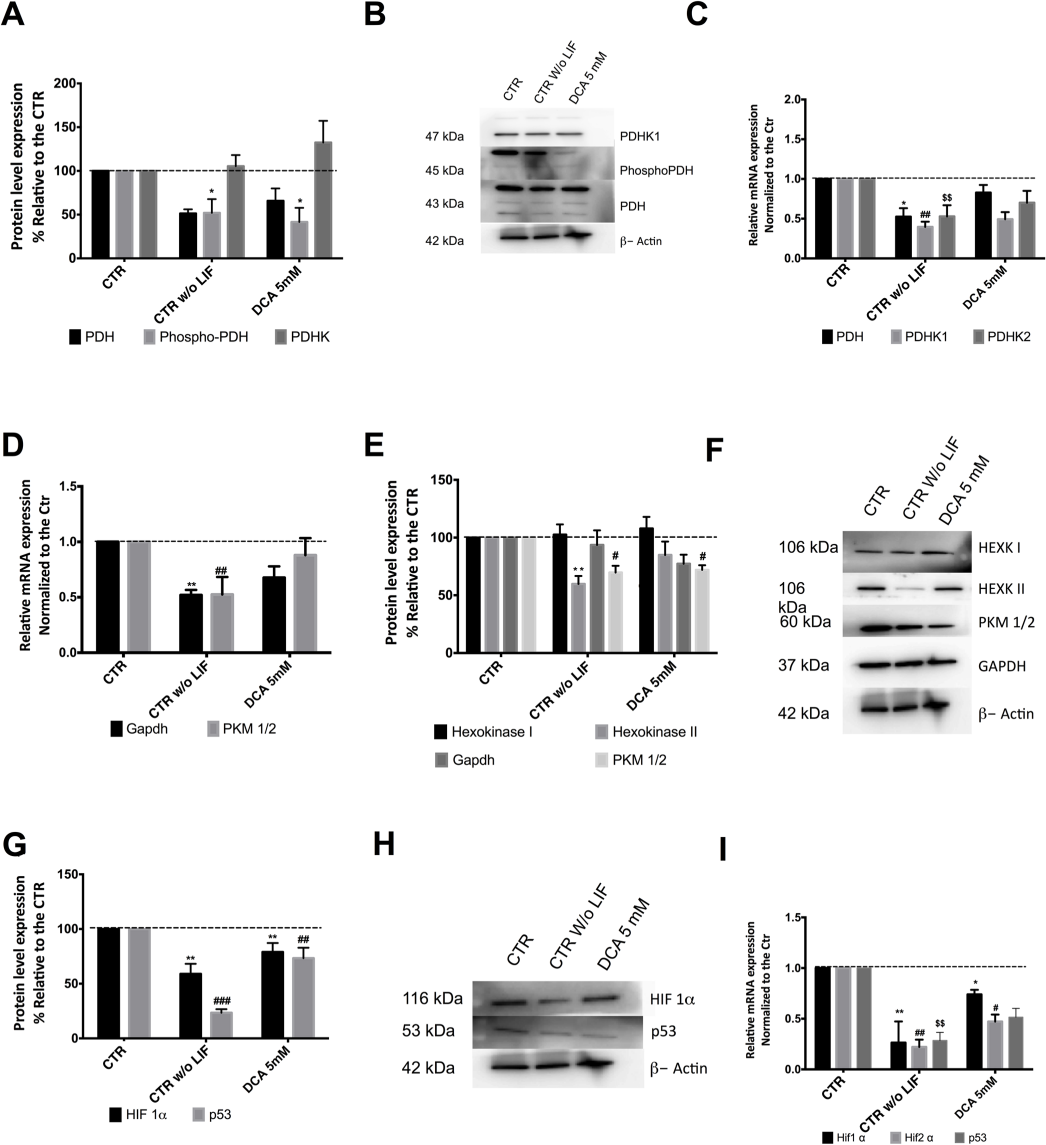
DCA effects on key proteins potentially involved in metabolic shifts. (A)- Protein levels for PDH, Phospho-PDH and PDHK1 were determined by western Blot and quantified by densiometric evaluation. Results were normalized to βeta-Actin levels and they are represented as percentage relative to the control; n = 4. A significant decrease in phosphorylated PDH (inactive form) was observed for CTR w/o LIF. (B)- Representative Western blot demonstrating the decrease in protein levels. (C)- qRT-PCR analysis for PDH, PDHK1 and PDHK2. Results are presented as fold changes and normalized to the reference house keeping gene βeta-Actin. Results represent 4 independent experiments. A significant negative impact was observed for all genes in the experimental condition CTR w/o LIF. (D)- qRT-PCR analysis for PKM1/2 and GAPDH. Results are presented as fold changes and normalized to the reference house keeping gene βeta-Actin. Results represent 3 independent experiments. Once more significant differences were observed for CTR w/o LIF. (E)- Protein levels for Hexokinase I and II, GAPDH and PKM1/2 were determined by western Blot and quantified by densiometric evaluation. Results were normalized to βeta-Actin protein levels and they are represented as percentage relative to the control; n = 4. PKM1/2 showed a significant decrease for all experimental conditions while for hexokinase II only CTR w/o LIF was significantly affected. (F)- Representative Western blot. (G)- Protein levels for Hif-1α and p53 were determined by western Blot and quantified by densiometric evaluation. Results were normalized to βeta-Actin protein levels and they are represented as percentage relative to the control; n = 4. Significant negative differences were observed for all experimental conditions. (H)- Representative blot. (I)- qRT-PCR analysis of Hif-1α, Hif-2α and p53. Results are presented as fold changes for 3 independent experiments, normalized to the reference house keeping gene βeta-Actin. * and # < 0.05; ** and ## p< 0.01; *** and ### p< 0.001 relative to the respective controls.

In a previous paper [8] we noted that pluripotency seemed to be accompanied by high levels of the phosphorylated form of PDH, so we wondered if DCA, which inhibits PDHK, was altering the PDH cycle, enhancing differentiation by activating PDH and shifting cells towards oxidative phosphorylation. Both the phosphorylated and non-phosphorylated forms of PDH, plus PDHK1, where analyzed by Western Blot and we observed a decrease in phosphorylated PDH (inactive form) and total PDH protein level, following DCA treatment and in the differentiation control (Fig 5A). Moreover mRNA levels mirrored protein results, with a significant negative impact for cells cultured in the absence of LIF (Fig 5C) for PDH (P<0.05); PDHK1 (P<0.01) and PDHK2 (P<0.01). This set of results points towards a more active PDH for cells grown without LIF and in the presence of DCA. Given that DCA is affecting pluripotency and the results suggests a shift in PDH regulation we assessed the protein levels for other key players in metabolic pathways.

Results for Gapdh and PKM1/2 (Fig 5D) suggest that only differentiating cells suffered a negative impact (P<0.01). In terms of protein levels, we observed that only hexokinase II levels decreased in cells grown without LIF when compared to the control (P<0.01) (Fig 5E). On the other hand, pyruvate kinase isoforms M1 and M2 (PKM1/2) showed a significant decrease for all experimental conditions (P<0.05).

Finally, given the particular importance of Hif-1α and p53 in metabolic shifts [1] we characterized protein (Fig 5G) and RNA levels (Fig 5I) for these possible targets under our experimental conditions. Interestingly differentiation (ESCs cultured without LIF) leads to a significant decrease in the levels of these proteins (P<0.01), paralleled by a decrease in their mRNA levels (P<0.01). The same was not true for cells exposed to 5mM DCA in the presence of LIF, given that changes for Hif-1α were less pronounced, and only protein levels significantly decreased for p53 (P<0.01), although a tendency was observed at the mRNA level. In general by differentiating cells (in the absence of LIF) or in the presence of 5 mM DCA protein levels for Hif-1α (P<0.001 and P<0.01 respectively) and p53 (P<0.05) significantly decrease.

## Discussion

PDHK can be considered an interesting gatekeeper in metabolism given that its activity determines the active or inactive state of PDH, resulting in variations of acetyl-CoA levels for the TCA cycle, which consequently determines the availability of substrates for mitochondrial OXPHOS [14,34]. In the normal glycolytic metabolism of ESC [8,35] an inactive (phosphorylated) form of PDH is preferred because this promotes the Warburg effect, with more rapid glucose turnover accompanied by more quiescent mitochondria. The available literature focusing on the inhibition of PDHK by DCA involves different types of medical conditions but is non-existent for ESCs (for review see [12]). Bearing in mind that this chemical compound is capable of inhibiting the four isoenzymes of PDHK and has been implemented in cancer research due to its collateral effect of promoting oxidative phosphorylation, cell death and cell cycle arrest [36] we wondered if its possible effect on PDHK might affect the pluripotent state of ESCs.

Our experimental approach determined that DCA, possibly via PDHK inhibition, clearly affected pluripotency in a similar manner to spontaneous differentiation, which was translated into differences in colony morphology as well as cell morphology. However, although there was a decrease in total cell number, partially explainable by viability results, this was not in agreement with proliferation rates. Our standard ESC culture conditions promote pluripotency, allowing a positive selection of pluripotent cells that will attach and grow more easily than differentiated ones. Thus it is reasonable to assume that cells that do attach are more proliferative when compared to control cells (which was indeed shown by PCNA), while non-attached/dead cells are removed. These results contradict the cell cycle arrest hypothesis and seem to confirm that DCA leads to cell death [37,38] even when ESCs are cultured with LIF.

In terms of pluripotency status, even though the absence of LIF seemed to be more detrimental, the results for AP staining and Oct4 as well as Nanog protein/mRNA levels clearly demonstrate a significant deleterious effect of DCA on pluripotency of ESCs grown with LIF. Furthermore, Nanog seemed to be less sensitive than Oct4 when a differentiation stimulus occurs. It is also important to note that although AP staining is commonly applied to assess pluripotency and constitutes an easy, fast assay [39], it is not the most robust pluripotency marker, and indeed the differences were not as significant as those found for the two key regulators of the pluripotency network, and were only significant for the experimental conditions that would promote a higher degree of differentiation.

Loss of pluripotency usually leads to an increase in mitochondrial membrane potential (MMP), which could confound DCA effects on ESC mitochondria, given that we did not observe the described DCA-induced decrease in MMP [12]. Indeed, inhibition of PDHK leads to a more oxidative metabolism that, in cancer cells, will result in increased apoptosis via the intrinsic mitochondrial pathway due to high ROS levels, causing mitochondrial depolarization and a decrease in ATP production [12,40]. Therefore, it is possible that DCA could have a particular impact in mESC when compared with other types of cells.

An inactive PDH (and functional PDHK) seem to be beneficial for pluripotency. When PDHK activity is negatively affected, possibly via DCA inhibition, lower levels of phosporylated PDH should be detected, and consequently more pyruvate would be available for the TCA cycle [41] which could be translated into higher oxygen consumption rates (OCR). Seahorse results showed that differentiated cells have a clear shift in metabolism when compared to control mESCs, with both a higher basal OCR consumption (data not shown) and generally a higher OCR throughout the assay. Consequently it is not surprising that differentiated cells exhibited higher coupling of oxidative phosphorylation to ATP production, with lower levels of proton leak, distinguishing themselves as the more metabolically active cells in this study. Although it has been described that DCA increases OCR [40], cells grown with DCA stood somewhat in between pluripotent and differentiated cells, with a clear metabolic shift only at the ECAR level.

Although, the results suggest an intermediate phenotype for cells treated with DCA, in this case there is a significant distance from pluripotent cells. Differentiating ESCs grown without LIF presented the lowest glycolytic flux, in agreement with the OCR results. With oligomycin addition we determined cell ability to rely solely on glycolysis to produce ATP and, interestingly, cells exposed to DCA seemed to better adapt to such conditions. This could be the reason why, when OCR was plotted with ECAR, cells grown with DCA were more “glycolytic”, probably meaning that DCA leads cells to be more metabolically malleable while pluripotent and differentiated cells rely more exclusively on glycolysis and OXPHOS, respectively. The differences in glycolytic capacity could reflect cellular adaptations resulting in an increase in glucose uptake, conversion of glucose to lactate instead of pyruvate, and glucose being used to produce metabolic intermediates for biosynthesis [40,42]. Scrutinizing which adaptations are operating in ESCs would be important in order to possibly control pluripotency/differentiation simply by changing media composition.

It is important to consider that metabolism is both a regulator of, and regulated by, signaling pathways. For this reason it is worthy to focus on possible signaling mediators already described for other models that share similarities with ESCs. Accordingly, it has been described that Hif-1α is upregulated in cancer cells, activating signaling pathways that will lead to an increase of PHDK1, PKM1/2 and HEXOKINASE II expression in order to promote a high rate of glycolysis [14,17,43,44]. To our knowledge there is no study demonstrating the same type of regulation in ESCs to maintain glycolytic metabolism, although it has been described that endogenous induction of pluripotency genes in induced pluripotent cells (iPSC) is preceded by a shift to glycolysis, with involvement of Hif-1α, PDHK and PKM [1,6,7,45]. It is important to highlight that in cells grown without LIF there is a decrease in Hif-1α, Hif-2α and p53 levels with concomitant changes in mitochondrial function and a decrease in enzymes known to be important in sustaining the glycolytic profile, such as Aldolase A, LDHA, PDHK1 and HKII. Curiously Hifs can be indirectly induced by succinate [46] and both have been implicated in the regulation of OCT4 [47]. Our results describe a similar decrease in the levels of those key factors in ESCs grown with LIF but in the presence of DCA. Notably, a decrease in Hexokinase levels was observed, and this could limit the first step of glycolysis compromising the maintenance of high glycolytic rates. Continuing to focus on glycolysis, we also observed a decrease in Gapdh levels and this enzyme controls the availability of substrates for the pentose phosphate pathway (PPP), which in turn is important to maintain NAPDH levels for the production of pentose moeities, both vital for rapidly dividing cells [48,49]. It is therefore tempting to consider that throughout loss of pluripotency the PPP becomes somehow impaired, something that would be interesting to confirm.

The significant decrease observed in PKM1/2 levels is also important, assuming that this enzyme has been implicated as a possible target for cancer therapeutics, by controlling the amount of pyruvate that is produced and additionally by shunting Glucose-6-Phosphate into the PPP. Also, PKM1/2 interacts with Hif-1α and Oct4 [50–52], which fits with the gene expression and protein level results profiled for both differentiating and pluripotent cells. This enzyme could therefore be involved in the maintenance of pluripotency, and thus could also be a potential target for metabolic modulation.

Considering that p53 controls cellular proliferation by inhibiting it if a cell is compromised, then p53 levels would de expected to drop in differentiating cells, as well as in cells grown in the presence of DCA [53–57], and this was indeed the case. It is important to consider that our results point towards similar pathways involved in controlling metabolism as those found in cancer cells, and that PDH could therefore constitute a good target for metabolic modulation, given its downstream effects.

Although other molecular biology strategies should be employed to dissect the exact role of PDHK in regulating ESC status, overall we can conclude that DCA promotes pluripotency loss and a shift in metabolism, meaning that under these conditions a putative inhibition of PDHK1 bypasses LIF, and this effect is very similar to differentiation in the absence of LIF. It is tempting to propose that if PDHK is inhibited, or in the presence of a signal that promotes differentiation, cells will adapt their metabolic regulatory circuitry in order to have a more active PDH. This adaptation will result in lower levels of Hif-1α, Hif-2α, p53, HKII, PDHK1 and PKM1/2, ultimately leading to more active mitochondria, and disrupting the glycolytic metabolic profile necessary to maintain pluripotency.
